# Paleomagnetic techniques can date speleothems with high concentrations of detrital material

**DOI:** 10.1038/s41598-022-21761-9

**Published:** 2022-10-26

**Authors:** Elisa M. Sánchez-Moreno, Eric Font, F. Javier Pavón-Carrasco, Luca A. Dimuccio, Claude Hillaire-Marcel, Bassam Ghaleb, Lúcio Cunha

**Affiliations:** 1 Faculdade de Ciências, Instituto Dom Luiz, Universidade de Lisboa, Lisboa, Portugal; 2 Departamento de Física, EPS, Universidad de Burgos, Burgos, Spain; 3 Departamento de Ciências da Terra, Faculdade de Ciências e Tecnologia, Universidade de Coimbra, Coimbra, Portugal; 4 Complutense University of Madrid, Madrid, Spain; 5 Geosciences Institute, IGEO, CSIC-UCM, Madrid, Spain; 6 Department of Geography and Tourism, Centre of Studies in Geography and Spatial Planning (CEGOT), FLUC, University of Coimbra, Coimbra, Portugal; 7 GEOTOP, Université du Québec à Montréal, Montreal, Canada

**Keywords:** Geophysics, Palaeomagnetism

## Abstract

The U-series dating of young and ‘dirty’ speleothems is challenging due to difficulties in assessing the isotopic composition of detrital contaminants and the low-abundance of ^230^Th generated in situ. Here we propose a new dating approach based on the comparison of a speleothem’s paleomagnetic directions to reference curves from global paleomagnetic reconstructions. This approach is demonstrated on a stalagmite collected from the Soprador do Carvalho cave in the Central Region of Portugal. A radioisotopic age model, built using four U-series ages and three ^14^C, suggests relatively steady carbonate precipitation from ~ 5760 BCE until ~ 1920 CE. Forty-five 6 mm-thick subsamples were analyzed using alternating field and thermal demagnetization protocols, providing well-defined, primary magnetic directions. An age model of the stalagmite was obtained by fitting its paleomagnetic record with the reference paleosecular variation curves obtained by previous paleo-reconstruction models, applying statistical bootstrapping analysis to define their best fit. The resulting age models fit closely with the radioisotopic age model but provide a significantly higher time resolution. We reach the same conclusion when applying this approach to another stalagmite from the Algarve region of Portugal. Our approach thus appears a promising alternative to date young speleothems with high detrital contents.

## Introduction

**Figure 1:**
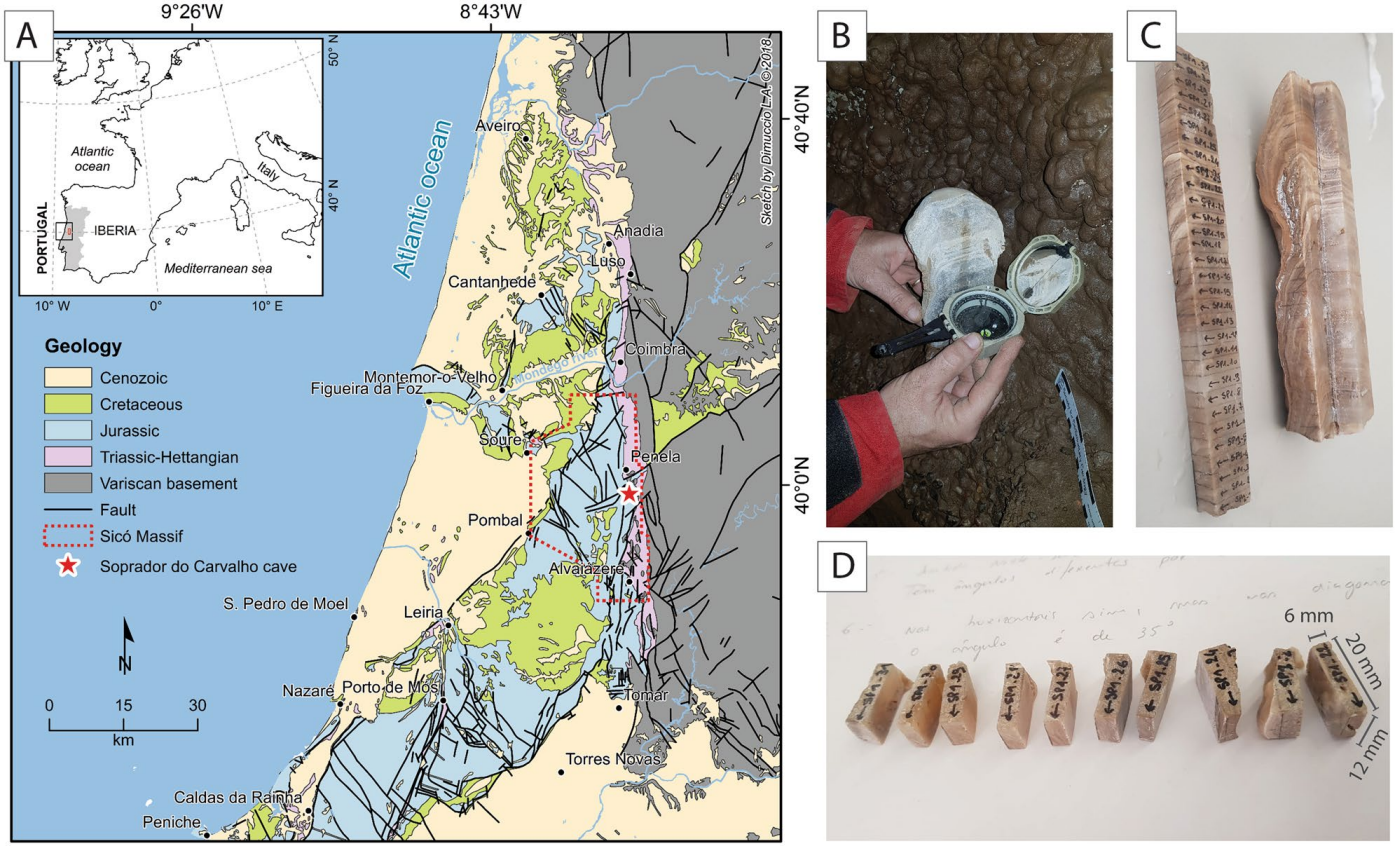
(**A**) Geological map of the studied area showing the location of the Soprador do Carvalho cave (map generated with the software ArcGIS Desktop 10.8.1 by ESRI). (**B**) The vertical front face of the half SP stalagmite was orientated in the cave by using a magnetic compass. (**C**) The half stalagmite was further cut into slices along the vertical axis, and subsequently, into (**D**) specimen of 2 cm × 1.2 cm × 0.6 cm.

Speleothems, defined as secondary mineral deposits formed in caves, are excellent recorders of climate and may have continuously recorded variations of the Earth’s magnetic field during their growth period. Their age can be determined precisely using U–Th series disequilibrium measurements, whereas magnetic, geochemical, and mineralogical signatures preserved in their thin laminations provide high-resolution climate- and environmental proxy time series at sub-annual to millennial time scales^1–8^. Speleothems host magnetic minerals that originate from the soils and rocks above a cave system. As these magnetic minerals are incorporated into actively growing stalagmites, the grains acquire a detrital remanent magnetization (DRM), which can accurately record the direction and relative intensity of the Earth’s magnetic field at deposition time and can be readily measured using standard superconducting quantum interference device (SQUID)-based rock magnetometers^9–18^. However, the use of speleothem as a paleomagnetic archive is paradoxical in the sense that “clean” (i.e., low detrital component) speleothems are generally excellent targets for U–Th radioisotopic dating but the acquisition of high-resolution paleomagnetic data is limited by the low concentration of magnetic minerals. Conversely, “dirty” (i.e., high detrital component) speleothems are better candidates for high-resolution paleomagnetic studies because they contain a considerable amount of magnetic minerals associated with the detrital fraction, but the determination of their precise U–Th age is hampered by the U and Th series isotopes linked to this fraction^19–21^. Pioneer workers compared magnetic declination and inclination from stalagmites with paleosecular variations of the Earth’s magnetic field together with geochronological data of speleothems^12,17,22^. Here, we propose to extend this approach to date dirty speleothems based on the recorded paleomagnetic information, more exactly by the fitting of paleomagnetic directional data along the calcite laminae with the Paleosecular Variation (PSV) curves derived from paleomagnetic reconstructions. Because available PSV models based on paleosecular variations cover only the last 14 kyr, older stalagmites cannot be dated using this approach. The fitting of paleomagnetic directional data with PSV curves has been achieved by using the *archaeo_dating* Matlab tool^23^, which is based on the combination of temporal probability density functions using paleomagnetic data only. We studied a stalagmite (labelled SP) from the Soprador do Carvalho cave, in the Central Region of Portugal, for which high detrital content is observable to the naked eyes by the brownish color of the calcite laminae (Fig. 1). We first dated the SP stalagmite based on U–Th isotopes to have an estimate of the time window during which the stalagmites developed, and to check for the reliability of the ages obtained by both methods. Finally, we discussed the limitations of our approach and the variability of the obtained ages by using different PSV models (SHA.DIF.14k^24^ and pfm9k^25^), and by studying another stalagmite from Southern Portugal^16,26^.

## Geological and geomorphological settings

The stalagmite studied (called SP) was collected in the Soprador do Carvalho cave (N 39° 59′ 9.91′′; W 8° 22′ 58.122′′), also known as the Talismã cave, in the Penela municipality (central-western Portugal). It is a horizontal cave of approximately 4000 m in length, which is crossed by an active underground river at its lower level^27–29^. The cave is located in the Sicó Massif^27,30^, in the sub-sector of Penela-Alvaiázere (Fig. 1), composed of a thick well-stratified, and generally continuous Jurassic carbonate succession^31,32^. In this sector, the largest endokarst system of the whole massif is the so-called “Dueça Speleological System”, which includes a ponor (Algar da Várzea), two temporary exsurgences (Gruta do Algarinho and Olho do Dueça) and two caves with mainly horizontal passages subject to seasonal flooding (Soprador do Carvalho and Brutiais)^27–29^. The Dueça Speleological system formed in the Middle Jurassic limestones that tectonically overlaps, at the local scale, the Lower Jurassic dolomitic-limestones (Sinemurian-Early Pliensbachian), with a remarkable decrease in the thickness of the interposed marl and marly-limestone units of the upper part of Lower Jurassic (Pliensbachian and Toarcian^32^). Hydrologically, this complex system currently allows the underground drainage of water from the plateau areas and valleys (at the elevation of ~ 250 m) towards the Olho do Dueça karst spring located at the elevation of ~ 200 m in the homonym Dueça River valley. In addition, some further ponors appear along this river, contributing to the hydrological recharge of the system.

The presence of a few generations of clastic cave sediments (that materialize siliciclastic fluvial terraces), in the Soprador do Carvalho cave^27,28^, point out a paleo-hydrological supply that involved the siliciclastic covers from the limestone plateaus and marly-limestone valleys to the west, as well as the erosion of schist hills to the east (in the outcrop domain of Variscan bedrock) (Fig. 1A).

The shallow depth at which the Dueça Speleological System was developed, its predominantly horizontal character, and the fact that the Soprador do Carvalho cave has a still-active hydrological functioning, suggest that speleogenesis is relatively recent.

Many of the speleothems from the carbonate massifs of Central Portugal are dirty due to the presence of a discontinuous Meso-Cenozoic siliciclastic cover that buried the Jurassic units, configuring the presence of a generally covered or partially exhumed (palaeo)karst^30,33^. In the Sicó Massif, this siliciclastic cover corresponds to polygenic red sands (with “Terra Rossa”) related to warm and arid climatic conditions that characterized the Paleogene-Neogene^27^.

## Results

### U–Th dating

Whereas detrital minerals incorporated into the calcite layers provide a means to achieve robust paleomagnetic property measurements, they reveal a challenge for the setting of ^230^Th-ages, especially in very young and U-poor stalagmites as illustrated in several studies^34,35^. Correction models for estimating U-series isotopes linked to the detrital fraction range from the assumption that this fraction bears a mean lithospheric isotopic signature^36^, to a modeled mean composition based on stratigraphical and coeval constraints^35^. As illustrated in Table 1, both approaches generally yielded poorly constrained ages. This is due to the relatively low precision achieved for ^230^Th measurements. As expected, the correction based on a “stratigraphically constrained” composition of the detrital fraction provides a more realistic age distribution than the empirical correction using the mean Earth’s lithosphere composition (Table 1). Nonetheless, both models imply a homogeneous isotopic composition of the detrital fraction in all samples analyzed, whereas magnetic properties of the study stalagmite rather suggest its grain size and mineralogical composition are variable, leading to also infer its variable U and Th concentrations and isotopic compositions.

**Table 1:** Radioisotopic data (^14^C and U–Th) of the Soprador do Carvalho stalagmite.

Sample	Depth (mm)	^14^C age	Cal. ^14^C age (yr BP)^a^		^238^U (ppb)	^232^Th (ppb)	AR(^234^U/^238^U)	^230^Th age (ka)^b^	Ludwig age	Roy-Barman age (yr)^b^	Isochron age (yr)^b^
		(% MC or yr BP)	Range	Median					(ka)^b^		
SP1-31	**1.5**	106.4 ± 0.4%	“Modern”	~ **1970**^**d**^	459.69 ± 1.28	25.40 ± 0.07	1.244 ± 0.005	3.18 ± 0.02	1.87 ± 0.67	1003 ± 542	109 ± 13^c^
SP1-29	15.5				423.01 ± 0.82	9.86 ± 0.02	1.228 ± 0.004	1.41 ± 0.01	0.85 ± 0.29	560 ± 455	
SP1-22	**57**	2013 ± 25 (UOC-16287)	**2002–1875**	**1955**	244.31 ± 0.66	33.05 ± 0.12	1.192 ± 0.004	7.64 ± 0.04	4.26 ± 1.74	1956 ± 1040	
SP1-19	74				132.30 ± 0.23	7.22 ± 0.01	1.179 ± 0.004	5.48 ± 0.03	4.11 ± 0.70	3173 ± 1146	
SP1-16	**95**	2646 ± 26 (UOC-16288)	**2784–2735**	**2755**	111.98 ± 0.30	2.43 ± 0.01	1.206 ± 0.004	3.63 ± 0.02	3.62 ± 0.02	2773 ± 231	
SP1-14	103.5				100.90 ± 0.20	3.63 ± 0.01	1.183 ± 0.004	4.95 ± 0.02	4.94 ± 0.02	3581 ± 893	
SP1-07	**147**	4405 ± 27 (UOC-16289)	5051–4865	4955	124.36 ± 0.33	17.76 ± 0.05	1.212 ± 0.004	9.93 ± 0.06	6.42 ± 1.80	4080 ± 1176	**4766 ± 269**^**c**^
SP1-06	152				134.23 ± 0.25	25.19 ± 0.05	1.195 ± 0.004	11.70 ± 0.06	7.00 ± 2.43	4397 ± 1323	
SP1-02	177.5				149.44 ± 0.25	3.78 ± 0.01	1.193 ± 0.004	6.30 ± 0.03	5.67 ± 0.32	5295 ± 611	
SP1-37	**178**				135.25 ± 0.71	1.61 ± 0.01	1.199 ± 0.007	5.74 ± 0.18	5.45 ± 0.23	5279 ± 221	**5249 ± 372**
SP2-12a	201				144.39 ± 0.24	1.47 ± 0.00	1.208 ± 0.004	5.95 ± 0.03	5.71 ± 0.13	5595 ± 383	
SP2-12b	**202**	5078 ± 28 (UOC-16290)	**5907–5745**	**5790**	193.67 ± 0.52	6.18 ± 0.02	1.228 ± 0.004	6.85 ± 0.04	6.09 ± 0.39	5630 ± 323	5525 ± 51
SP2-01	**267**	6888 ± 28 (UOC-16291)	**7792–7664**	**7710**	136.80 ± 0.36	45.61 ± 0.12	1.203 ± 0.004	22.53 ± 0.12	14.14 ± 4.35	8531 ± 3640	

^a^95.4% probability; Year 0 = 1950 CE.

^b^Year 0 = the measurement year 2020 CE.

^c^2-point isochrons built using two samples from close depths.

^d^Estimate from the model discussed in an appendix.

Bold characther ages: anchor ages for the radioisotopic age model.

To better estimate the isotopic composition at the level of the interval sampled for U-series and ^14^C measurements, attempts at setting two-point isochrons were made, either by analyzing two powder fractions from the sample, when enough material was available or, by combining measurements in two vertically close samples when this revealed impossible. Four of such isochrons could be set (at depths of 1.5 mm; 147–152 mm; 177.5–178 mm; 201–202 mm). Results are also reported in Table 1. They suggest nearly-continuous and steady carbonate precipitation at least since ~ 5.5 ka, likely since ~ 8 ka anchoring the stalagmite bottom age with the calibrated ^14^C age, both providing a first estimate of the mean sedimentation rate (~ 0.034 mm·year^−1^).

### Radiocarbon ages

The ^14^C ages reported in Table 1 were calibrated using the IntCal20 calibration curve^37^, assuming an isotopic equilibrium between the cave CO_2_ and the atmospheric CO_2_, aside from the isotopic fractionation that can be monitored by the current ^13^C-based isotopic normalization^38^. The tight linkage between ^230^Th-isochron ages and the ^14^C calibrated age suggests that the cave CO_2_ remained close to equilibrium with atmospheric CO_2_ with respect to its ^14^C activity despite the complex mechanisms regulating the isotopic composition of cave CO_2_^2^ (for a Portuguese karst system).

The core top sample activity (~ 106% vs “modern carbon”) indicates the presence of some thermonuclear carbon in the sample, thus at least part of this sample was deposited after 1950 CE. This observation also supports near-equilibrium conditions between the cave CO_2_ and its dissolved inorganic carbon, vs the atmospheric CO_2_, at least within measurement error bars. The effective age of the core top is discussed in the Supplementary Information section. A simple model based on steady-state conditions during the deposition of the 3 mm-thick top sample would suggest a stalagmite growth ending by ~ 1972 CE.

A very tight fit between calibrated ^14^C ages and ^230^Th-derived ages is observed at the stalagmite top, at ~ 147–152 mm, and 201–202 mm (Table 1). They further support the assumption of a 14C/12C near-equilibrium between the cave CO_2_ and atmospheric CO_2_. We thus added three calibrated ^14^C ages as complementary anchors at ~ 57, 95, and 272.5 mm (Table 1), to derive an age model. The “anchor ages” used were thus: ~ 1972 CE (end of precipitation); ~ 5 BCE (57 mm); ~ 805 BCE (95 mm); ~ 2746 BCE (149 mm); ~ 3229 BCE (178 mm); ~ 3840 BCE (202 mm); ~ 5760 BCE (272 mm). Unfortunately, estimating potential errors on these anchor ages is uneasy, as their calibrated ^14^C ages should be seen, in principle, as maximum ages within the uncertainties of the corresponding ^230^Th-constrained age. However, the good fit between the two sets of ages led us to hypothesize that the uncertainty of the ^14^C calibrated age would encompass any age error linked to a small ^14^C-offset between the atmospheric CO_2_ and the cave CO_2_ that could have occurred back in time. Minimum and maximum ages retained for each anchor age are thus those defined by the probabilistic distribution of the corresponding calibrated ^14^C age or, at depths where such ages were not available, on the uncertainty of the ^230^Th-isochron age. As the calibrated ages retained are the median of their probabilistic distribution following the calibration and because ^230^Th- and ^14^C-calibrated uncertainties are combined at some depths, the offsets between the ages retained and their minimum and maximum values are unequal.

### IRM acquisition curves

**Figure 2:**
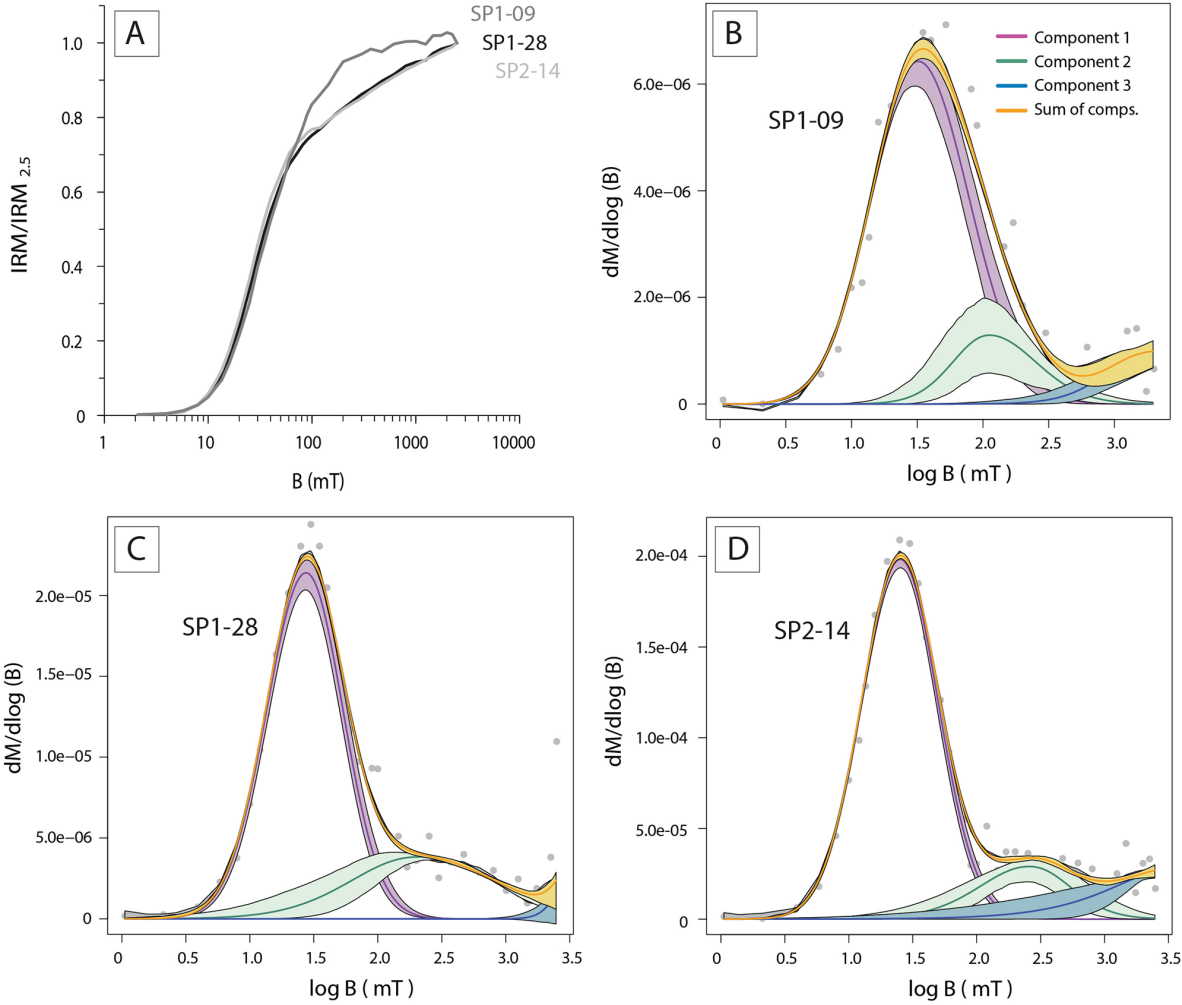
(**A**) Normalized isothermal remanent magnetization (IRM) acquisition curves of the SP stalagmite. (**B–D**) Skewed generalized log-Gaussian function of representative SP samples obtained with the MAX UnMix software showing the presence of three magnetic components, assigned to be magnetite, hematite, and goethite, respectively.

Analysis of the Isothermal remanent magnetization (IRM) acquisition curves by the skewed generalized (log) Gaussian functions with the Max UnMix software^39^ has led to the identification of three main magnetic components, for which representative samples are illustrated in Fig. 2. For all the studied specimens, the skewness of the IRM curves is negligible and the data better fit a log-normal distribution. Component 1 is the dominant magnetic phase with a contribution of the total remanence of ~ 80%, with B_1/2_ values comprised between 25 to 34 mT and DP of ~ 0.30, typical of detrital and/or pedogenic magnetite^40^. Component 2 contributes to ~ 15% of the remanence and shows values of B_1/2_ (~ 250 mT) and DP (~ 0.40) typical of hematite. Component 3 ~ contributes to ~ 5% of the total remanence and has B_1/2_ values of ~ 2000 mT, with DP of ~ 0.30, which can correspond to high coercivity hematite and/or superparamagnetic goethite^41,42^.

### Paleomagnetism

**Figure 3:**
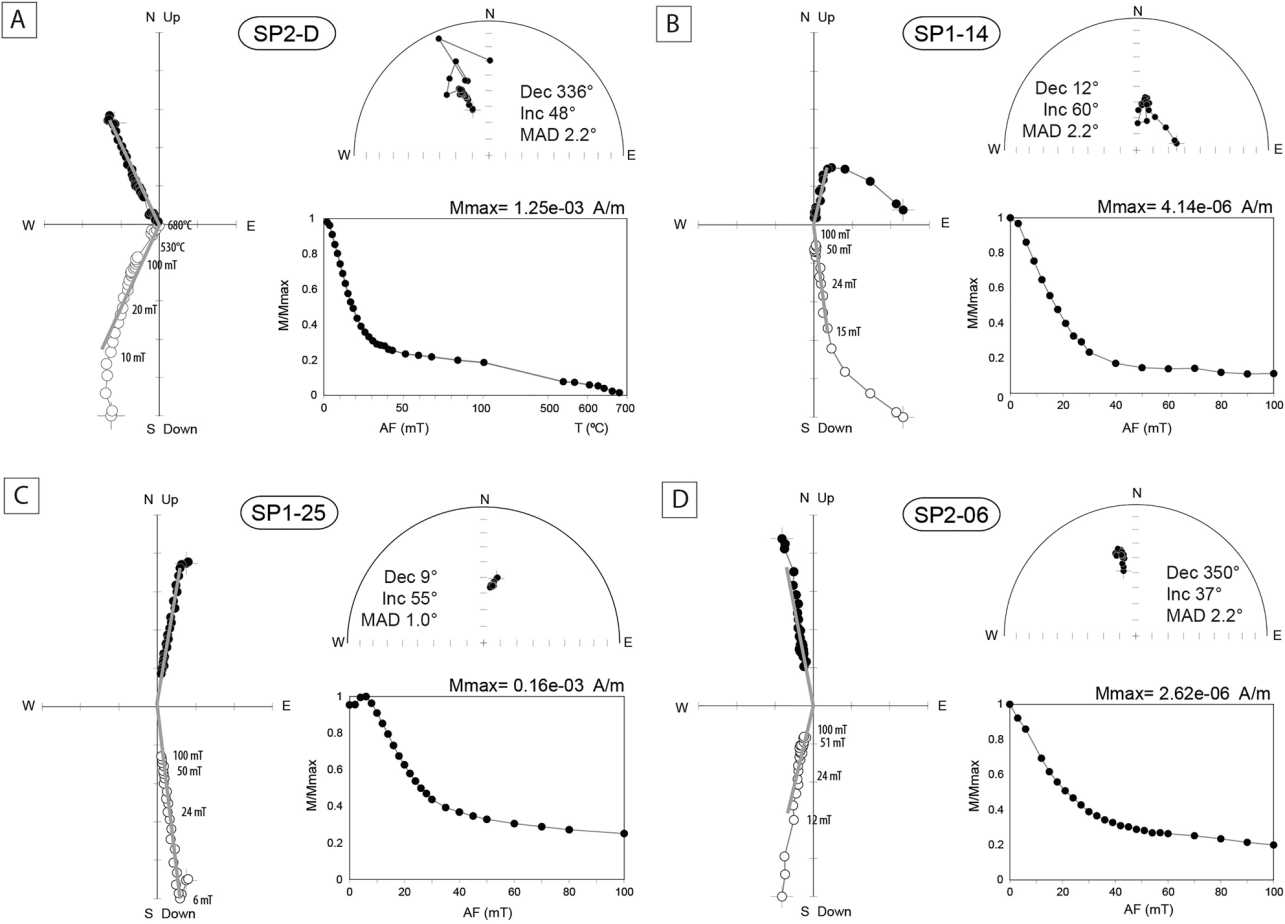
Stereographic and orthogonal projections and remanence intensities versus alternating field (AF) and temperature of representative SP samples. Grey lines in the orthogonal plot represent the vector of the characteristic remanent magnetization (ChRM).

We first measured the remanent magnetization per unit volume of pilot samples to evaluate the minimum thickness required to be measurable on the 2G cryogenic magnetometer. Pilot samples have relatively high NRM intensities, on the scale of 10^–3^ A/m, which allows to measure small plate-like samples of ~ 6 mm in thickness (Fig. 1C,D). Paleomagnetic measurements were then performed on 45 samples. NRM intensities vary from 2.52 × 10^–4^ to 3.09 × 10^–2^ A/m with an average of 5.03 × 10^–3^ A/m. After AF demagnetization, 43 samples provide stable and reliable magnetic vectors, whereas two samples give erratic demagnetization vectors (sample SP1-08 and SP1-09) (Fig. 3, Table 2). A viscous remanent magnetization (VRM) is observed in most samples and is removed by demagnetization fields of 3–12 mT (Fig. 3). Characteristic remanent magnetization (ChRM) vectors pointing to the origin of the orthogonal plot are isolated between 6 and 100 mT. Median destructive fields (MDF) range from 10 to 40 mT, typical of magnetite (Fig. 3). In most samples, 70 to 90% of the natural remanence is removed after demagnetization at 100 mT, suggesting that a hard coercivity magnetic mineral (hematite) is carrying part of the remanence. The remaining 10–30% is cleaned by using thermal demagnetization between 530 and 680 °C (in 5 pilot samples) and shows unblocking temperatures of ~ 680 °C, typical of hematite (Fig. 3). Magnetic directions carried by magnetite are well clustered and show positive magnetic inclinations of ~ 30°–60°, with declination varying from W 25° to E 22° (Fig. 3). The magnetization carried by hematite shows similar directions, albeit with more scattered and erratic directions, probably due to the noise associated with the weak intensity of the remanence at these levels.

**Table 2:** Paleomagnetic data and age model results for Soprador do Carvalho speleothem (each paleomagnetic direction corresponds to n = 1).

Sample	Pmag results						Age-depth models (CE/BCE)			
	Depth (mm)	AF_min_ (mT)	AF_max_ (mT)	Dec (°)	Inc (°)	MAD (°)	SHA.DIF.14k		pfm9k	
							Age	Error age	Age	Error age
SP1-31	3	8	100	350.2	49.8	3.3	1443	84	1472	119
SP1-30	9	4	100	352.0	39.7	3.6	1347	56	1380	78
SP1-29	15	8	100	340.4	43.7	2.3	1251	51	1296	56
SP1-28	21	14	100	343.1	50.1	2.2	1139	48	1217	48
SP1-27	27	8	80	341.7	49.7	4.3	1037	40	1175	51
SP1-26	33	4	100	334.6	59.7	1.2	982	36	1154	55
SP1-25	39	6	100	9.0	54.7	1.0	972	37	1119	59
SP1-24	45	6	100	5.1	47.4	0.8	949	37	1049	69
SP1-23	51	8	80	18.6	35.4	2.1	846	48	925	80
SP1-22	57	8	100	17.1	45.0	3.3	645	69	740	95
SP1-21	63	9	100	4.9	53.6	1.3	377	91	528	108
SP1-20	69	15	90	359.6	57.7	2.6	95	102	335	117
SP1-19	75	9	90	1.3	51.6	3.2	− 163	98	160	127
SP1-18	81	9	80	356.6	49.2	4.1	− 398	90	− 32	138
SP1-17	87	6	80	5.3	49.1	3.2	− 631	81	− 267	143
SP1-16	93	18	70	355.6	53.2	6.0	− 830	73	− 524	131
SP1-15	99	21	40	345.2	48.6	5.5	− 946	67	− 753	127
SP1-14	105	15	70	11.5	59.5	2.2	− 1006	63	− 939	143
SP1-13	111	18	70	21.7	57.4	3.8	− 1088	69	− 1107	145
SP1-12	117	9	27	77.0	52.3	6.1	− 1243	85	− 1288	141
SP1-11	123	6	100	6.8	55.4	2.3	− 1445	113	− 1483	148
SP1-10	129	6	100	5.0	56.5	3.8	− 1631	152	− 1670	167
SP1-09	135	–	–	–	–	–	− 1783	180	− 1850	180
SP1-08	141	–	–	–	–	–	− 1926	194	− 2040	179
SP1-07	147	6	80	0.9	54.6	3.1	− 2100	193	− 2259	165
SP1-06	153	6	80	2.7	49.2	3.1	− 2332	174	− 2512	154
SP1-05	159	6	100	349.9	58.5	2.2	− 2600	144	− 2776	157
SP1-04	165	6	90	353.4	43.5	2.6	− 2835	122	− 3006	166
SP1-03	171	6	100	352.8	50.5	3.3	− 2999	109	− 3187	187
SP1-02	177	6	90	350.7	58.3	2.2	− 3108	98	− 3346	195
SP1-01	181.5	9	70	356.4	52.8	3.5	− 3180	94	− 3474	174
SP2-14	187.5	40	100	359.9	48.7	3.4	− 3292	97	− 3682	133
SP2-13	195	21	100	358.2	46.6	1.9	− 3465	106	− 3983	104
SP2-12	201	15	100	356.6	42.9	1.0	− 3613	116	− 4174	92
SP2-11	207	15	100	342.9	45.9	1.8	− 3751	129	− 4273	93
SP2-10	213	6	100	347.8	48.0	1.6	− 3876	138	− 4310	100
SP2-09	219	6	100	349.7	47.1	1.3	− 3982	142	− 4324	103
SP2-08	225	9	100	351.0	46.3	1.5	− 4069	135	− 4340	95
SP2-07	231	12	100	351.4	40.0	2.2	− 4149	112	− 4371	79
SP2-06	237	12	100	350.1	37.3	2.2	− 4248	93	− 4426	66
SP2-05	243	6	90	352.9	35.4	3.4	− 4409	99	− 4512	62
SP2-04	249	6	100	358.2	33.1	1.7	− 4675	120	− 4634	71
SP2-03	255	6	100	5.6	38.6	2.6	− 5073	138	− 4806	97
SP2-02	261	6	100	7.1	46.0	1.7	− 5592	177	− 5040	140
SP2-01	267	12	100	359.4	49.1	1.4	− 6307	312	− 5388	198

Error ages are given at 95% of probability.

*Sample* name of the measured specimen, *Depth* depth of the central point of each sample, *AF*_*min*_* − AF*_*max*_ minimum and maximum alternating field steps selected to calculate paleomagnetic direction (i.e. ChRM, characteristic remanent magnetization), *Dec* magnetic declination, *Inc* magnetic inclination, *MAD* maximum angular deviation of paleomagnetic direction, *CE* Common Era, *BCE* Before Common Era, *SHA.DIF.14k* ages obtained by fitting SP paleomagnetic data to the paleosecular variation (PSV) curve from the SHA.DIF.14 k global reconstruction^24^, *pfm9k* ages obtained by fitting SP paleomagnetic data to the PSV curve from the pfm9k global reconstruction^25^.

### Paleosecular variation age dating

**Figure 4:**
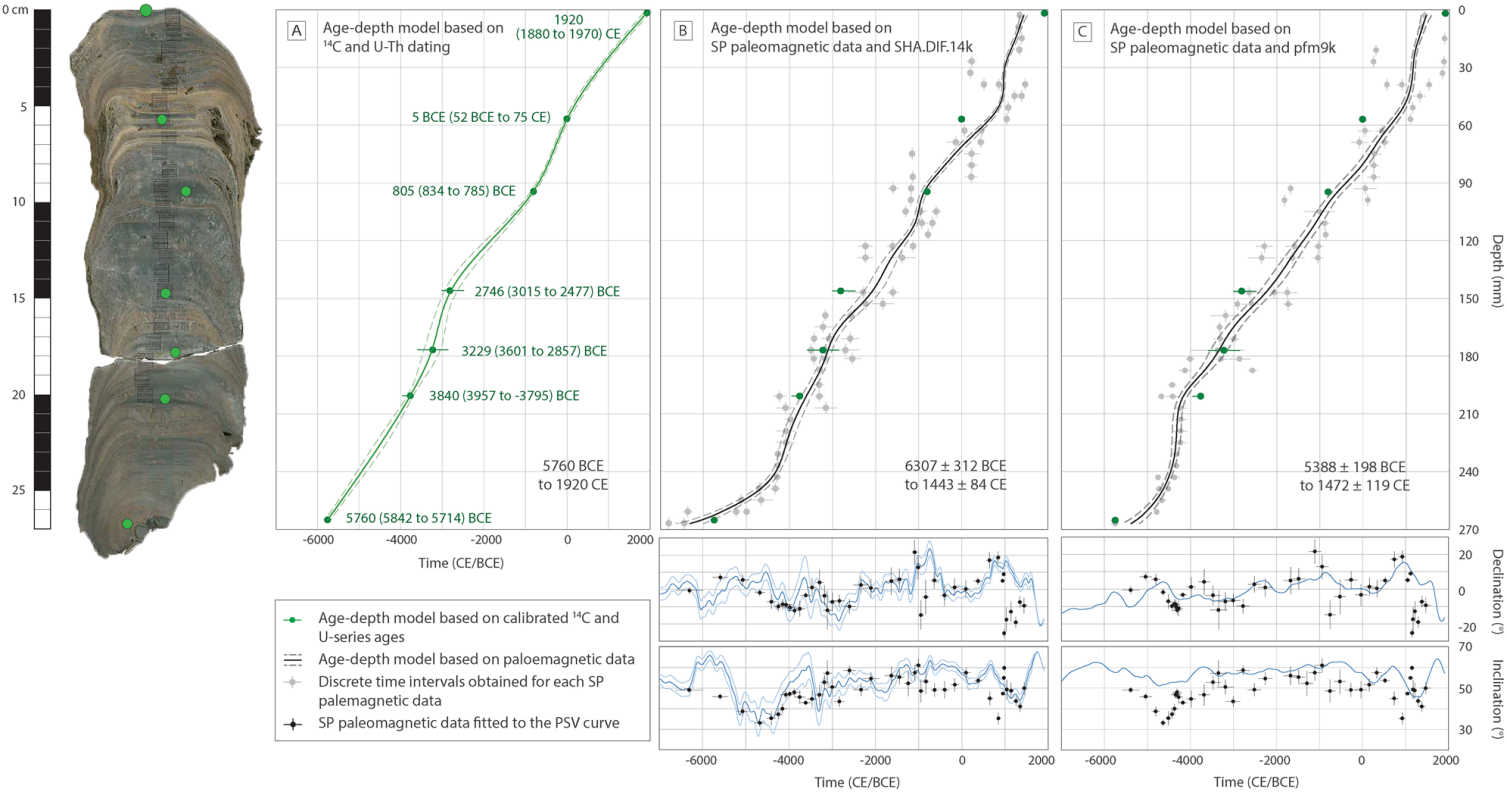
Age-depth models of the Soprador do Carvalho stalagmite. (**A**) Age-depth model based on calibrated ^14^C and U–Th anchor ages. (**B**) Age-depth model (*above*) obtained after fitting its paleomagnetic data to the magnetic declination and inclination of the PSV curve of the SHA.DIF.14k model (below). (**C**) Equal to (**B**) but using the PSV curve of the pfm9k model. Grey points on the upper panel of (**B,C**) correspond to the different dating intervals given by using the *archaeo_dating* tool (see Table S1 of the Supplementary Information). Vertical error corresponds to half of the specimen thickness (± 3 mm), while horizontal error corresponds to the 95% confidence interval of dating according to the *archaeo_dating* outputs. Black points on the lower panels in (**B,C**) correspond to the paleomagnetic directions calculated for each SP specimen at the ages estimated by the corresponding age model (the vertical error is the maximum angular deviation associated with the characteristic remanent magnetization (ChRM), and the horizontal error correspond to the age errors given by the paleomagnetic age models). Calibrated ^14^C and U–Th dating (green points) are illustrated in (**B,C**) for comparison. Maximum (bottom of the stalagmite) and minimum (top) ages for each age model are indicated by the black text. All age models are represented with error bands at 95% of probability.

We compare the paleomagnetic directions (declination, inclination along with the directional angular error estimated by the maximum angular deviation) obtained from the 43 samples with PSV models from the same geographic location by using the *archaeo_dating* Matlab tool^23^. The objective is to get the best fit between our paleomagnetic data and the past geomagnetic field direction. According to the radioisotopic dating based on U–Th isotopes, the SP speleothem covers the last 9 ka, for which different PSV models are available, including the CALS10k.2 and ARCH10k.1 models^43^, the pfm9k^25^, and the SHA.DIF.14k models^24^. The CALS10k.2 and pfm9k models provide smooth variations of the past Earth’s magnetic field because the database includes paleomagnetic directions from sediments, which may suffer from inclination shallowing. For this reason, we opted to use here the SHA.DIF.14k model (Fig. 4B), which is based on archaeomagnetic and volcanic data only. Despite the smoothing problems in the models cited above, we also use the pfm9k model to test the reliability of our approach (Fig. 4C). The ARCH10k.1 model was also developed using only archaeomagnetic and volcanic data and does not provide error bars for the PSV curves, which is a key point in the dating approach. For our study, we choose a fixed time window of 7000 BCE to 1900 CE.

The *archaeo_dating* Matlab tool allows for the dating of archeological and geological objects by comparing the paleomagnetic directions measured in the samples with a master PSV curve based on the combination of temporal probability density functions^23^. However, because of the recurrent pseudo-periodicity of the past geomagnetic field, multiple solutions are possible for each sample. To circumvent this issue, we used the principle of stratigraphic superposition, i.e., the dating intervals for a given sample cannot be older than the dating intervals obtained in deeper samples. In addition, the ratio of sample depth versus the calculated age, which should be represented by a smooth quasi-linear curve, serves as an additional criterion for the selection of the dating intervals (see Fig. S2 of the Supplementary Information). The resulting dating intervals obtained for the samples are illustrated by grey dots with error bars (at 95% of probability) in Fig. 4B,C using the SHA.DIF.14k and pfm9k models, respectively. The error in depth associated with each sample corresponds to half of the thickness of the paleomagnetic samples (i.e., 3 mm).

The next step consists in fitting the dating intervals as a continuous function of the sample depth. We used a Monte Carlo method based on a random bootstrap approach, where each dating interval (for the same depth) is sampled following a homogeneous distribution with the dating limits given by the *archaeo_dating* tool (grey dots in Fig. 4B,C). The use of a homogeneous distribution relates to the fact that ages estimated within the dating interval have the same level of probability^23^. It is worth noting that the error in depth has also been considered in the bootstrap approach using a normal distribution. A first random bootstrap selection (following the two previous distributions) provides a set of age-depth pairs of data that are next fitted to a continuous curve using penalized cubic b-splines (see also Supplementary Information for more information about the fitting approach). This procedure is repeated several times to get an ensemble of age-depth curves. The penalized cubic b-splines require establishing an optimal value of the so-called damping parameter (see Eq. (1) in the Supplementary Information). The selection of the optimal value of the damping parameter depends on the distribution and dispersion of the data. This parameter is chosen considering the group of trade-off curves of the data misfit (given as the root mean square error) for different values of damping parameters. The mean trade-off curve presents a clear knee point close to the optimal value of the damping parameter (see Fig. S3 of the Supplementary Information). Higher values than the optimal parameter provide smoother variations for the age models (almost a linear fitting), in contrast, lower values generate unrealistic variations for the age model due to the dispersion of the paleomagnetic dates. After establishing the optimal value of the damping parameter, the bootstrap is repeated 10^6^ times before to obtain the final ensemble of age-depth curves, for which mean (black line in Fig. 4B,C) and standard deviation (dashed grey lines in Fig. 4B,C, which represent twice standard deviations, i.e. at 95% of probability) provide the final age model. The ages of the bottom and top of the stalagmite interpolated from the age model from SHA.DIF.14k are 6307 ± 312 BCE and 1443 ± 84 CE, respectively (5388 ± 198 BCE and 1472 ± 119 CE, using the pfm9k model).

## Discussion

Speleothems are remarkable recorders of paleoclimatic conditions as long as their thin calcite laminae can be precisely dated. Conventional geochronological methods used in speleothems include ^14^C and U–Th radioisotopic dating^1^. The radiocarbon method is limited to the last 0.5–50 ka (i.e., about 9 half-life of ^14^C of ~ 5700 year) and requires corrections for the proportions of dead carbon issued from the dissolution of the carbonate bedrock^44–46^. Due to recent analytical advances in mass spectrometry, U-Th dating provided accurate chronological constrains of speleothem for the past 500 ka, even with low ^238^U concentrations^1,47,48^. However, U-Th dating of “dirty” speleothems, those containing significant detrital components incorporated in the matrix, is problematic due to the contribution of detrital U-series isotopes^20–22^. Diagenetic alterations, including aragonite-to-calcite transformation and calcite-to-calcite recrystallization^49^, may also severely compromise the accuracy of the U–Th chronology by mobilizing U from the site of diagenesis and leading to an increase in the ^230^Th/^238^U isotopic ratio^50–52^. For these reasons, many dirty speleothems are discarded despite they may provide important paleoclimate and paleoenvironmental information recorded in the detrital fraction.

Paleomagnetism offers an interesting alternative for dating dirty speleothems, but such an approach has not been explored hitherto. The principle is based on the capability of magnetic minerals, principally magnetite, issued from the overlying soil/sedimentary cover and carbonate bedrock to settle down in the calcite laminae and align according to the direction of the Earth’s magnetic field. Once calcite precipitation is completed and has trapped the magnetic minerals, magnetization locks in almost instantly^53^. This process provides several advantages in using paleomagnetic directions as a dating tool over U–Th methods, including the fact that magnetic minerals are commonly more resistant to diagenetic alteration than calcite; magnetic measurements are cost-effective, allowing a much more complete and detailed database; and is particularly suitable for dirty speleothems, which exhibit high remanent magnetization due to the relatively high concentration of magnetic particles contained in the detrital fraction. Unlike sediments, the magnetization recorded in speleothem is not supposed to be affected by inclination flattening due to compaction, as long as samples are taken in the central part of the speleothem, in the horizontal layers, where particle rolling due to gravity is minimum^16^. Because U–Th is usually expensive, many age-depth models of speleothem are built on a few dating points, with depth/age interpolation using the StalAge software^54^, assuming a nearly constant growth rate between each U–Th datings. However, growth rates are rarely constant, and this can result in poorly constrained age-depth models (Fig. 4A). Conversely, paleomagnetism can fill this gap by providing a higher resolution dataset. The thickness of the samples depends on how dirty the speleothem is, i.e., on the number of magnetic particles trapped in the calcite laminae to be measurable in a standard magnetometer. Pioneer paleomagnetic studies of speleothem usually used 2 cm cubic samples^11,12,14,17^. Advances in magnetometer sensitivity now allow to measure the magnetic remanence of speleothem at a millimetric scale, typically of some millimeters (6 mm in this study) up to the centimeter^10,16,26,55,56^, providing high-resolution records of the past Earth’s magnetic field.

The main objective of this study is to test whether a speleothem can be accurately dated based on the comparison of the recorded paleomagnetic directions with a well-known paleomagnetic database. This approach requires that: (i) the magnetization is primary and well-defined by principal component analysis; (ii) magnetic inclination shallowing is negligible or minimum; (iii) the time window in which the speleothem grew is approximatively known; and that (iv) well constrained PSV curves do exist for this time window (coming from global or local paleo-reconstructions).

Analysis of the isothermal remanent magnetization (IRM) curves of the studied samples indicates that detrital/pedogenetic magnetite is the main magnetization carrier (Fig. 2). A small contribution of hematite is observed in the IRM curves and in the demagnetization intensity decay curves, where only 80% of the remanence is cleaned after AF demagnetization at 100 mT (Fig. 3). Thermal demagnetization up to 700 °C shows that the magnetic directions carried by hematite are comparable to those carried by magnetite, suggesting that this hematite is detrital as well. Well-defined and stable demagnetization patterns pointing to origin (Fig. 3) also attest to the primary character of the remanent magnetization.

Ponte et al. demonstrated that some speleothems experience dramatic magnetic inclination shallowing depending on the slope of the calcite layers. This bias is interpreted as the result of particles rolling and the gravity effect when magnetic particles are transported along the borders of the stalagmite. In the case of the SP stalagmite, we collected samples along the central growing axis, where calcite layers are horizontal and where magnetic inclination should be minimized. However, the central growing axis may be dislocated during the formation of the stalagmite and some samples can include significantly inclined calcite layers, as is the case on the left border of the samples SP-17 to SP-23 (Fig. 1C). However, no significant magnetic inclination shallowing has been noted in these samples compared to the over- and underlying horizontal layers, suggesting that if magnetic inclination occurred in these samples, it should be minimum or negligible, probably because the inclined layers represent a small percentage of the total volume of the sample.

Because most of the available Holocene paleomagnetic models are limited to the last 10,000 to 14,000 year (the CALS10k.2, the ARCH10k.1, the pfm9k, and the SHA.DIF.14k models), only speleothems younger than 14,000 year can be dated with our approach. The first step is to define the time window into which paleomagnetic data are compared to the reference PSV curve. This can be done by dating the base and the top of the stalagmite, but more U-Th dating can be added to confirm and calibrate the final age-depth model obtained by using the two different approaches (Fig. 4). Here we obtained the age model illustrated in Fig. 4A based on the seven U-series and calibrated ^14^C anchored ages indicated in Table 1. Based on these results we consider the time interval of 2000 CE to 7000 BCE for both SHA.DIF.14k and pfm9k models provide alternative age-depth models based on paleomagnetic data only (Fig. 4B,C), following the approach described in Sect. 4.4. The resulting age-depth model from SHA.DIF.14k provides slightly different but overlapping age intervals than those obtained with U-series and calibrated ^14^C ages, except for the base and the top of the stalagmite (Fig. 4B). We obtained minimum and maximum ages of 6307 ± 312 BCE and 1443 ± 84 CE, respectively. The shape of the age-depth model using paleomagnetic data exhibits more details and inflections due to a higher number of datapoint (43 paleomagnetic samples against 7 anchored radioisotopic ages). These inflections should reflect changes in the stalagmite growth rates. The misfit between the age of 1920 CE measured for the more recent calcite layer at the top of the stalagmite and the age of ~ 1440 CE provided by our approach can be explained by the presence of a significant gap in the calcite precipitation rate at the most external (younger) part of the stalagmite, as shown in Fig. S1 of the Supplementary Information. The age of 1920 CE is obtained from the most superficial calcite layers, whereas the last (younger) paleomagnetic specimen has been sampled just below (some mm). The misfit between the ages of the base of the stalagmite remains, however, unresolved.

Our method provides an interesting and promising approach to date dirty speleothem based on paleomagnetic data but has its limitations. First, we assume that the master PSV curve used for comparison is correct and robust, which is not necessarily the case for all time intervals. The differences observed in the PSV models proposed in the literature depend on several factors: the type of data used for their construction (i.e., igneous rock, sediments, archeological artifacts) and their spatial and temporal distributions; the dating and measurement uncertainties associated with the different samples and how they are treated in the modeling approach; the statistical analysis used to develop the models; etc. As mentioned above, the CALS10k.2^43^ and the pfm9k^25^ models include all kinds of paleomagnetic data around the world, including paleomagnetic data from sediments. This results in PSV curves with smoother magnetic inclination than the SHA.DIF.14K model, which includes paleomagnetic data from archeological objects and volcanic rocks only. This smoothness is reflected in the local inclination minimum presented in the SHA.DIF.14k curve around 4000–5000 BCE that is not recorded by the pfm9k model (see inclination curves in the bottom panels of Fig. 4B,C). It is important to note that the time interval from 5000 BCE to 1900 CE is relatively well-document in terms of paleomagnetic data^57^, whereas some time intervals lack critically reliable paleomagnetic data (ex. from 7000 to 5000 BCE).

**Figure 5:**
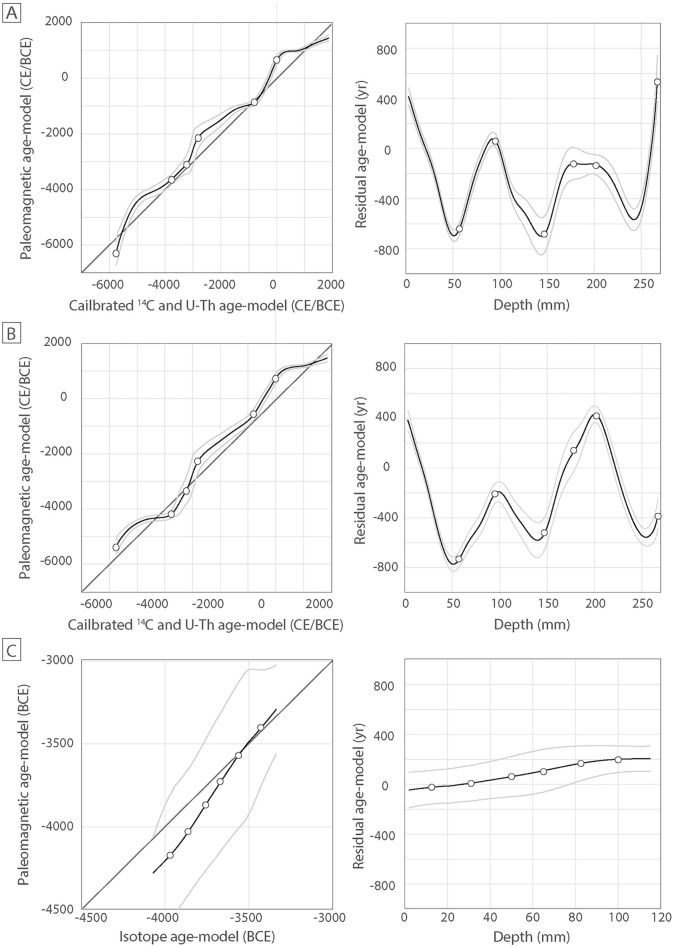
Correlation between the age-depth model obtained by calibrated ^14^C and U-series isochrons (cf. Fig. 4A) and the model based on the comparison between the SP paleomagnetic data and the PSV curves from (**A**) the SHA.DIF.14k model, and (**B**) the pfm9k model (cf. Fig. 4B,C). Opened circles correspond to the calibrated ^14^C and U–Th dating (see Table 1). The confidence intervals are calculated based on the paleosecular variation age model only and are plotted at 95% of probability. (**C**) Equal correlation performed on SPAIV speleothem^26^ with paleomagnetic data fitted to the PSV curve obtained with SHA.DIF.14k.

We also tested our approach with the pfm9k family model^25^ to evaluate how different the results are depending on the choice of the PSV master curve. We used the pfm9k.1a model with the error bars given by the model version 1b (version 1a does not provide error bands). We first synthesized a directional PSV curve for the speleothem coordinates and obtained a new age model by fitting its paleomagnetic profiles to the pfm9k curves illustrated in Fig. 4C. The resulting age-depth model is closely similar to those calculated with the SHA.DIF.14k model and with radioisotopic data, with a minimum and maximum age of 5388 ± 198 BCE and 1472 ± 84 CE, for the base and the top, respectively. Interestingly, the younger age provided by using the SHA.DIF.14k or the pmf.9k model are strikingly comparable within the 95% probability interval. The older age (base) is younger and still statistically distinct from the age calculated based on radiocarbon analysis, but with lower residual values than those calculated when using the SHA.DIF.14k model (Fig. 5).

Figure 5 illustrates the correlation and residual values from the mean (y = x line in Fig. 5) between the age-depth model calculated with radioisotopic ages and the age-depth model calculated based on paleomagnetic data using the SHA.DIF.14k and the pmf.9k PSV curves. We discarded the ^14^C age of 1920 CE of the most external layer due to the presence of the hiatus (Fig. S1) interpreted to be responsible for the misfit with the age of ~ 1440 CE calculated with our approach. We observe a striking correlation when using both the SHA.DIF.14k and pmf.9k models. The exception is the age determined at the base of the stalagmite, where the correlation gives a residual value of ~ 500 year for the SHA.DIF.14k model and a residual value of ~  − 400 year for the pmf.9k model. We interpreted this discrepancy since both models are poorly defined before 4000–5000 BCE.

**Figure 6:**
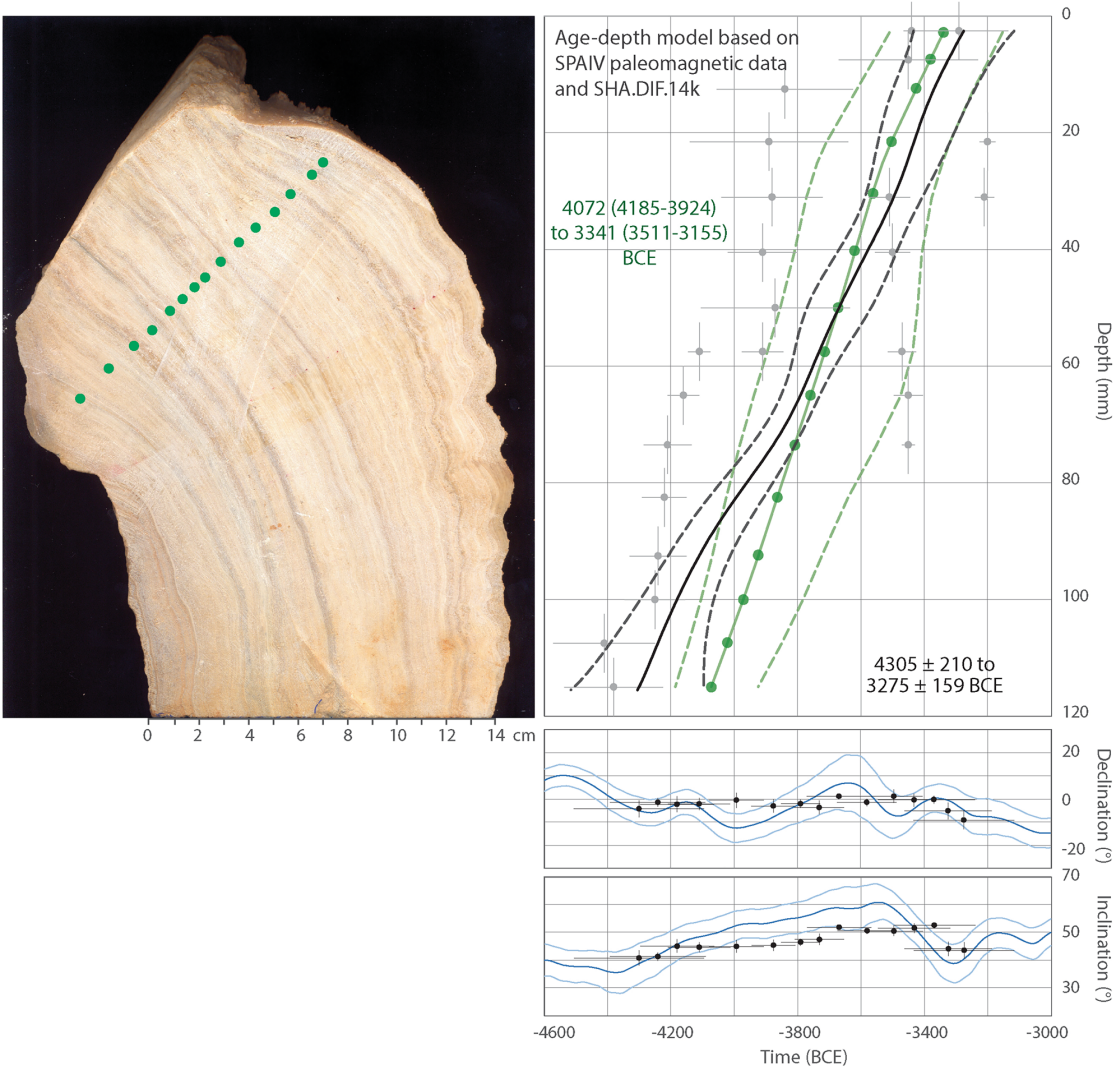
The age-depth model of the Algarve speleothem^16,26^ obtained by fitting the paleomagnetic data to the PSV curve from the SHA.DIF.14k model, using our statistical bootstrapping analysis (in black). Age-depth model based on interpolated U-series ages (in green). Below is the position of the paleomagnetic data on the PSV curve after fitting. See Fig. 4 for legend. Green points in the speleothem picture represent the sample-based mean directions calculated by Ref.^26^ in 15 calcite laminae for which ages were calculated based on U-series isochrons using the StalAge algorithm. All data errors and error bands are shown at 95% of probability.

We also test our approach in the case of another stalagmite from the Algarve area^16,26^. This stalagmite (called SPAIV) is a middle-Holocene dirty stalagmite that formed during the ~ 4100–3300 BCE interval, based on U-series isochron ages (Fig. 6). The age model of this stalagmite was calculated based on 6 U-series isochron ages, and intermediate ages corresponding to each paleomagnetic site-based. The paleomagnetic site-based data corresponds to the mean of the directions obtained from several specimens collected in the same calcite layers. The interpolated ages were obtained using the StalAge algorithm^54^. In this case, we have used the SHA.DIF.14k paleo-reconstruction to constrain its age model. As in the case of the Soprador do Carvalho stalagmite, the age model calculated with paleomagnetic data is closely comparable to the U-Th age model, within their overlapping 95% confidence intervals (Fig. 6). Residual values between both set of data are lower than 200 year (Fig. 5C).

## Conclusion

In conclusion, speleothems with high detrital content can be accurately dated based on the comparison of paleomagnetic data with paleosecular variation models for the last 14,000 year. Application of the *archaeo_dating* Matlab tool, combined with a Monte Carlo method based on a random bootstrap approach, to the paleomagnetic data of the Soprador do Carvalho and SPAIV stalagmites, provided detailed age-depth models, which are statistically similar to the age model obtained by conventional calibrated ^14^C and U-series dating. Our approach opens new perspectives to overcome the limitations inherent to the radioisotopic dating of dirty speleothems, providing opportunities to include them in future paleoclimatic and paleoenvironmental reconstructions.

## Methods

### Sampling

The stalagmite SP understudy was extracted from the cave and was subsequently cut in the laboratory into two pieces along the vertical plane. Later, we came back to the cave and positioned one-half part of the stalagmite in the original position to orientate the front face using a magnetic compass (Fig. 1B). The measured azimuth and dip of the vertical section are N 80° and 90°, respectively. Sub-sampling for paleomagnetic measurements was subsequently made in the laboratory by cutting individual small plate specimens of approximately 20 × 12 × 6 mm, leading to a total of 45 specimens (Fig. 1C,D).

### U-series dating

U-series isotope dating was performed at the Radiochronology laboratory of the GEOTOPO-UQAM-McGill research Center (Canada). Thirteen subsamples were collected in the 29 cm-long stalagmite, each from a 3 to 6 mm-thick cut perpendicular to the growth axis of the stalagmite, to recover from 0.4 to 1.9 g of carbonate: the minimum quantity needed to obtain the precision required to estimate the ^230^Th fraction from the in situ decay of ^234^U. The thickest cut was made for the youngest surface sample, expected to depict very low contents in the in-situ produced ^230^Th.

Each subsample was ground in an agate mortar for subsequent U-series isotope measurements. The sample powders were dissolved using nitric acid in a Teflon™ beaker into which a weighted amount of calibrated mixed spike ^233^U–^236^U–^229^Th had been placed and evaporated slowly to dryness. After the dissolution, around 10 mg of the iron carrier was added and the solution was then left-over night for spike-sample equilibration. U and Th were coprecipitated with Fe(OH)_3_ by adding ammonium hydroxide drop by drop until reaching pH 8–9. The precipitate was recovered by centrifugation and washed twice with deionized water, then dissolved in 6 N HCl. U–Th separation was performed on a 2 mL AG1X8 anionic resin volume. The thorium fraction was recovered through elution with 6 N HCl, and the U and Fe fraction, with water. The U fraction was purified on a 0.2 mL U-Teva™ (Elchrom industry™) resin volume. Fe was eluted with 3 N HNO_3_ and the U fraction with 0.02 N HNO_3_. After drying, thorium purification was carried out on a 2 mL AG1X8 resin in 7 N HNO_3_ and eluted with 6 N HCl. U–Th measurements were performed using a multi-collector inductively coupled plasma mass spectrometry Nu instrument™. ^236^U–^235^U–^234^U–^233^U and ^232^Th–^230^Th–^229^Th were measured on the ion counter (IC0) in peak switching mode for uranium and thorium isotopes, respectively. ^238^U was not measured but calculated from ^235^U/^236^U ratios, assuming a constant ^238^U/^235^U mass ratio (137.88). Knowing ^236^U/^233^U of the spike, mass bias corrections in the atomic mass unit (amu^−1^) were calculated and used to correct measured ratios between uranium isotopes and between thorium isotopes. After total evaporation, 2 mL of HCl supersaturated with H_3_BO_4_ were added, and the solution was evaporated to dryness, before re-dissolution with HCl 6 M. The overall analytical reproducibility was estimated from replicate measurement of a uraninite standard Hu-1. Precision is usually better than ± 0.5% for uranium results and around 1% for thorium in young samples, both at two sigma levels.

### Radiocarbon measurements and calibration

Aliquots of the homogenized powder prepared for U-series analysis were used for ^14^C measurements. They were processed and graphitized as described in Crann et al. Measurements were performed at the A.E. Lalonde AMS Laboratory of Ottawa (Canada) on a 3MV tandem accelerator mass spectrometer built by High Voltage Engineering. The ^12,13,14^C^+3^ ions were measured at 2.5 MV terminal voltage with Ar-stripping. The fraction vs modern carbon, F^14^C, was calculated according to Ref.^58^ as the ratio of the sample ^14^C/^12^C ratio to the OXII standard ^14^C/^12^C ratios^59^ measured in the same data block. Both ^14^C/^12^C ratios were background-corrected, and the result was corrected for spectrometer and preparation fractionation using the AMS-measured ^13^C/^12^C ratio normalized to δ^13^C =  − 25‰ (VPDB). Radiocarbon ages in “Libby’s years” were calculated as − 8033ln(F^14^C) and reported in ^14^C yr BP (BP = AD 1950; sensu^38^). Errors on ^14^C ages (± 1σ) were based on counting statistics and ^14^C/^12^C and ^13^C/^12^C variation between data blocks. Calibration of ^14^C ages into the CE/BCE chronology was performed using the IntCal20 Northern Hemisphere Radiocarbon Age Calibration Curve of Ref.^37^.

### Isothermal remanent magnetization

Samples were first cleaned by alternating field (AF) demagnetization up to 100 mT. Isothermal remanent magnetization (IRM) was conducted by applying ~ 40 pulse steps up to 2.5 T in samples SP2-14, SP1-09, and SP1-28. IRM was imparted with the IM-10–30 impulse magnetizer (ASC scientific) and remanence was measured with a 2G magnetometer of the Paleomagnetism Laboratory of the University of Burgos (Spain). IRM acquisition curves were unmixed into several components based on skewed generalized (log) Gaussian functions with the Max UnMix softwatre^39^ to isolate the contributions of magnetite, hematite, and goethite (noted here as components 1, 2, and 3, respectively). Each component is characterized by the values of the saturation IRM (SIRM), the mean applied field to reach half of the saturation (B_1/2_), and the dispersion parameter of the Gaussian curve (DP).

### Paleomagnetism

Magnetic measurements were performed at the Paleomagnetism Laboratory of the University of Burgos (Spain) with a superconducting cryogenic magnetometer (2G Enterprises) coupled with an AF device. A stepwise AF demagnetization protocol was applied up to 100 mT with 16 to 24 consecutive AF demagnetization steps. Characteristic remanent magnetization (ChRM) was calculated using Principal Component Analysis (PCA)^60^ and Fisher statistics with the Remasoft 3.0 software. In addition, thermal demagnetization (TH) of 5 pilot samples was performed from 530 up to 680 °C with a TD48-DC (ASC) oven at the Paleomagnetism Laboratory of the University of Burgos.

## Supplementary Information

Supplementary Information.

## Data Availability

Correspondence and requests for data should be addressed to E. M. S-M (emsanchez@ubu.es).
