# Supplementary material for: Paleomagnetic techniques can date speleothems with high concentrations of detrital material
Source: Sci Rep. 2022 Oct 26;12:17936. doi: 10.1038/s41598-022-21761-9 (PMC9605962; doi:10.1038/s41598-022-21761-9)
Supplement: Supplementary file 1 — Supplementary Information. [file 41598_2022_21761_MOESM1_ESM.docx]

*Supplementary information for*

Paleomagnetic techniques can date speleothems with high concentrations of detrital material

Elisa M. Sánchez-Moreno^1,2*^, Eric Font^1,3^, F. Javier Pavón-Carrasco^4,5^, Luca A. Dimuccio^6^, Claude Hillaire-Marcel^7^, Bassam Ghaleb^7^, Lúcio Cunha^6^

^1^Universidade de Lisboa, Instituto Dom Luís (IDL), Faculdade de Ciências, Lisboa, 1749-026, Portugal

^2^Universidad de Burgos, EPS, Departamento de Física, Burgos, 09006, Spain

^3^Universidade de Coimbra, Departamento de Ciências da Terra, Faculdade de Ciências e Tecnologia, Coimbra, 3000-272, Portugal

^4^Complutense University of Madrid, Madrid, 28040, Spain

^5^Geosciences Institute, IGEO, CSIC-UCM, Madrid, 28040, Spain

^6^University of Coimbra, Centre of Studies in Geography and Spatial Planning (CEGOT), FLUC, Department of Geography and Tourism, Coimbra, 3004-530, Portugal

^7^Université du Québec, GEOTOP, Montreal, H2X 3Y7, Canada

* [emsanchez@ubu.es](mailto:emsanchez@ubu.es)

**1. Stalagmite top age**

The SP stalagmite was not active when it has been collected. However, the ^14^C activity of the aliquot from the 3 mm-thick top sample used for U-series measurements (+63.82±3.49 ‰ vs Modern Carbon) indicates the presence of some carbonate precipitated following the thermonuclear experiments. A simple model can be used to estimate the age structure of the top 3 mm of the stalagmite, assuming that:

1. equilibrium conditions of ^14^C between the cave CO_2_ and dissolved inorganic carbonate vs atmospheric CO_2_ prevailed through time without any significant lag;
2. precipitation was continuous and with a steady annual rate of ~ 0.034 mm/yr as suggested by the overall age structure of the stalagmite, leading to infer a ~ 87 yr time span recorded in the 3 cm top sample.

Then, the ^14^C activity of the carbonate sample would be the sum of 1/87 of the mean annual ^14^C activity of atmospheric CO_2_, over the 87 floating years. Using the atmospheric CO_2_ record of Hua et al. (2013) and Graven et al. (2017), and iterative calculations, the interval covered by the stalagmite top sample would then represent the ~ 1885-1972 CE interval, with a mean age set at ~ 1920 CE. The relatively robust U-series age of this sample (109±13 yr), with a measurement made in 2020 CE, fits well with the ^14^C-model derived mean age of this sample.

However, a strong discrepancy is observed between the ^14^C age of 1920 yr CE and the age of ~1450 yr CE estimated from the paleomagnetic approach (Fig. 4) for the younger sample (SP1-31 in Fig. S1). As shown in Figure S1, the gap of -500 yr could be explained by the presence of a hiatus between the SP1-31 sample and the top layer of the stalagmite, which can be interpreted as a period of non-deposition or calcite dissolution.

**
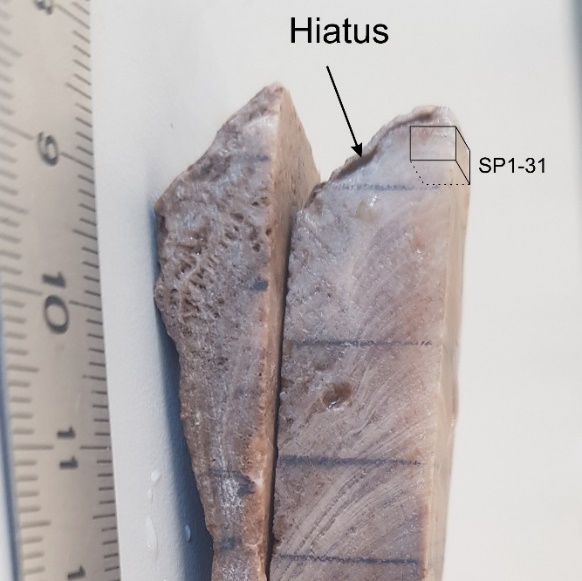
**

**Figure S1.** Photograph of the top of the SP stalagmite showing the presence of a gap in the calcite precipitation.

**2. Age model based on paleomagnetic information**

Paleomagnetic directional data of the stalagmite SP have been dated by comparing the declination and inclination angles with a directional paleoosecular variation (PSV) curve of the past Earth’s magnetic field at the same location of the speleothem. This comparison is carried out using the Matlab tool *archaeo_dating*^3^ and two different PSV curves synthesized by the SHA.DIF.14k^4^ and pfm9k^5^ paleo-reconstructions. Using this Matlab tool, we start dating the sample located at the top of the speleothem and we obtain a possible time interval for the last 1000 yr. of about 1380 ± 45 CE and 1435 ± 65 CE when using the SHA.DIF.14k and pfm9k.1 models, respectively. The first SHA.DIF.14k dating interval is obtained from the probability density function (PDF) represented as a red curve in Figure S2.

We then apply the same procedure to the second sample. It is important to note that for this second sample, we constrain the *a priori* dating interval using a similar time range (last 1000 yr) but slightly shifted into older times (see oblique black lines in Figure S2). We apply this temporal constraint since two consecutive samples should have similar dating intervals (the age model must be a smooth curve, as it can be seen in the obtained age model based on the isotope dating, Figure 4A). The consecutive dating for all of the SP’s samples provides a set of PDF curves covering the last 9 kyr. (see red curves in Figure S2). Finally, the Matlab tool estimates for each PDF curve the most probable age of the speleothem sample at 95% of confidence. These most probable ages are represented as grey dots (with error bars at 95% of confidence) in Figure 4 of the main text.

**
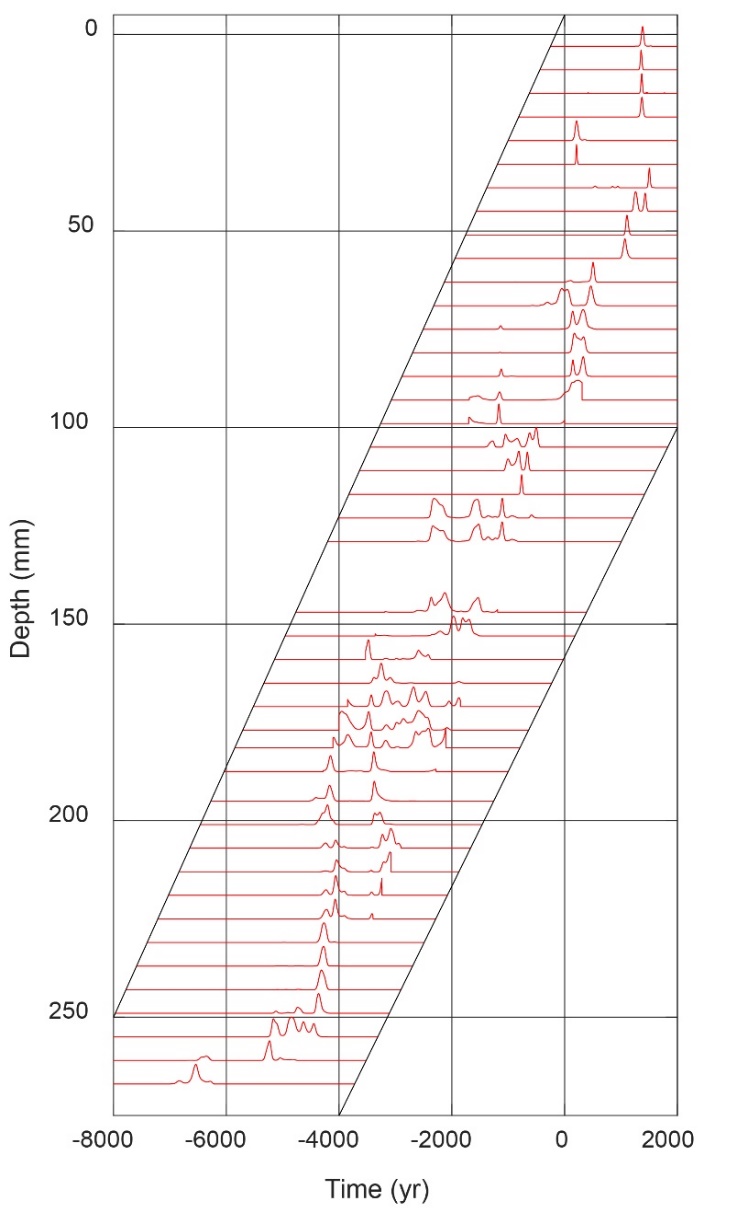
**

**Figure S2.** Probability density functions (PDF) of the archaeomagnetic age for each speleothem sample according to the SHA.DIF.14k^4^ paleo reconstruction. The PDF's are given by the Matlab tool *archaeo_dating*^3^ and are delimited by a time interval following the criteria detailed in the manuscript.

Once we get the most probable dating intervals for each sample (see Figures S2, 4b-c, and 6), we apply a bootstrap method to fit all the pair of data “age-depth” into a smooth curve, i.e., the paleosecular variation age model. To do that, we consider two different distributions for the age intervals and the depth (see Method of the main text). Then, we randomly select a pair of “age-depth” for each sample following the previous distributions and we fit those points considering penalized cubic b-splines with knot points every 6 mm from the top (0 mm) to the bottom (267 mm) of the SP speleothem. For each random pair of data, we get the age model as:

$f\left( d \right)=\left( B^{'}\cdot B+\lambda\cdot c \right)^{-1}B^{'}\cdot d$ [1]

where $B$ is a matrix based on the b-cubic splines for each knot point and at the different depth data; $d$ is a vector with the age data; and $R$ is a penalization matrix that provides smooth curves according to the damping parameter $\lambda$.$B^{'}$ is the transpose matrix of $B$. The obtained age model curve is represented by $f\left( d \right)$.

We carry out different tests to estimate the optimal value of $\lambda$. Figure S3 shows the outcome for different $\lambda$ values in terms of rms-misfit. The mean trade-off curve presents a clear knee point close to the optimal value of the damping parameter and it was fixed to as $\lambda$ = 10. Note that the selection of the optimal damping parameter depends on the distribution and dispersion of the data. In our case, the chosen damping parameter provides smooth variations for the age-models (realistic models) and then, a major part of the points falls outside the age-model uncertainty (see Figure 4B-C of the main text) because we are fitting the trend data not the set of individual data. If we modify the damping parameter to get a more fitted age model, unrealistic variation can appear due to the dispersion of the paleomagnetic dates. With the chosen optimal damping parameter, we repeat our bootstrap approach 10^6^ times to get an ensemble of age model curves. The final age model is given by the mean values of the ensemble (see black lines in Figure 4B-C of the main text). The final error bands for each age-model are estimated by calculating the standard deviation of the ensemble and they are represented at 95% of probability (see dashed black lines in Figure 4B-C of the main text).


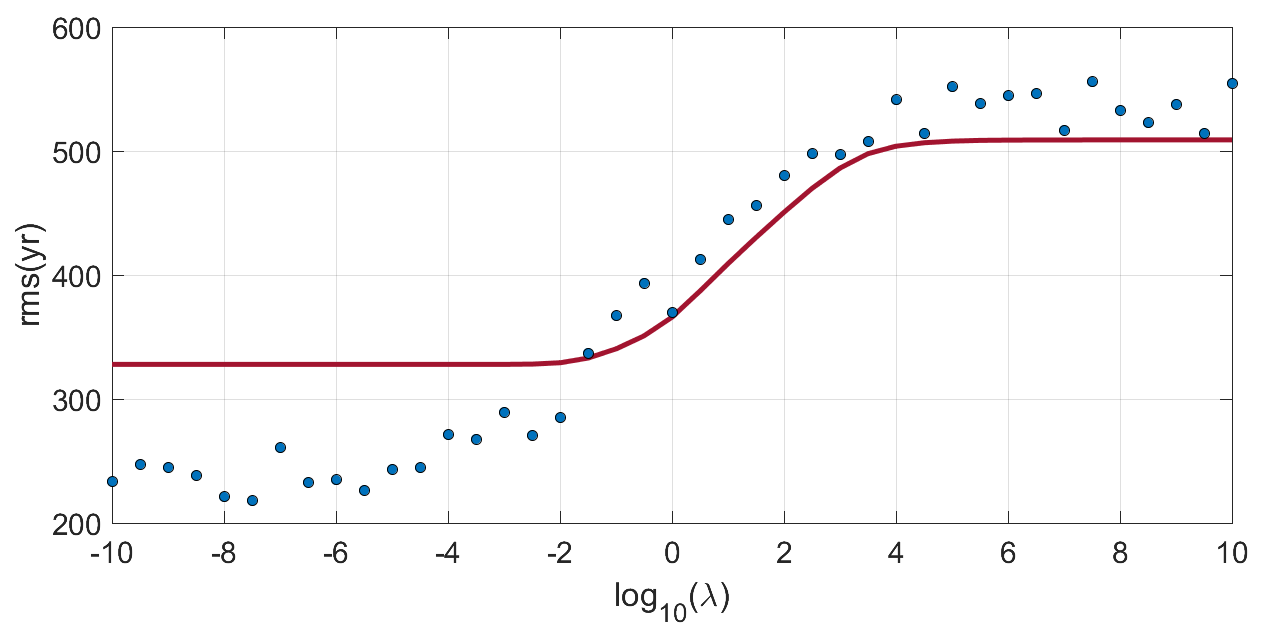


**Figure S3**. Trade-off curve using different $\lambda$ values. Blue dots are the mean rms-misfit for the different random datasets (considering the error data) and the red line is the test when no error is considered.

**
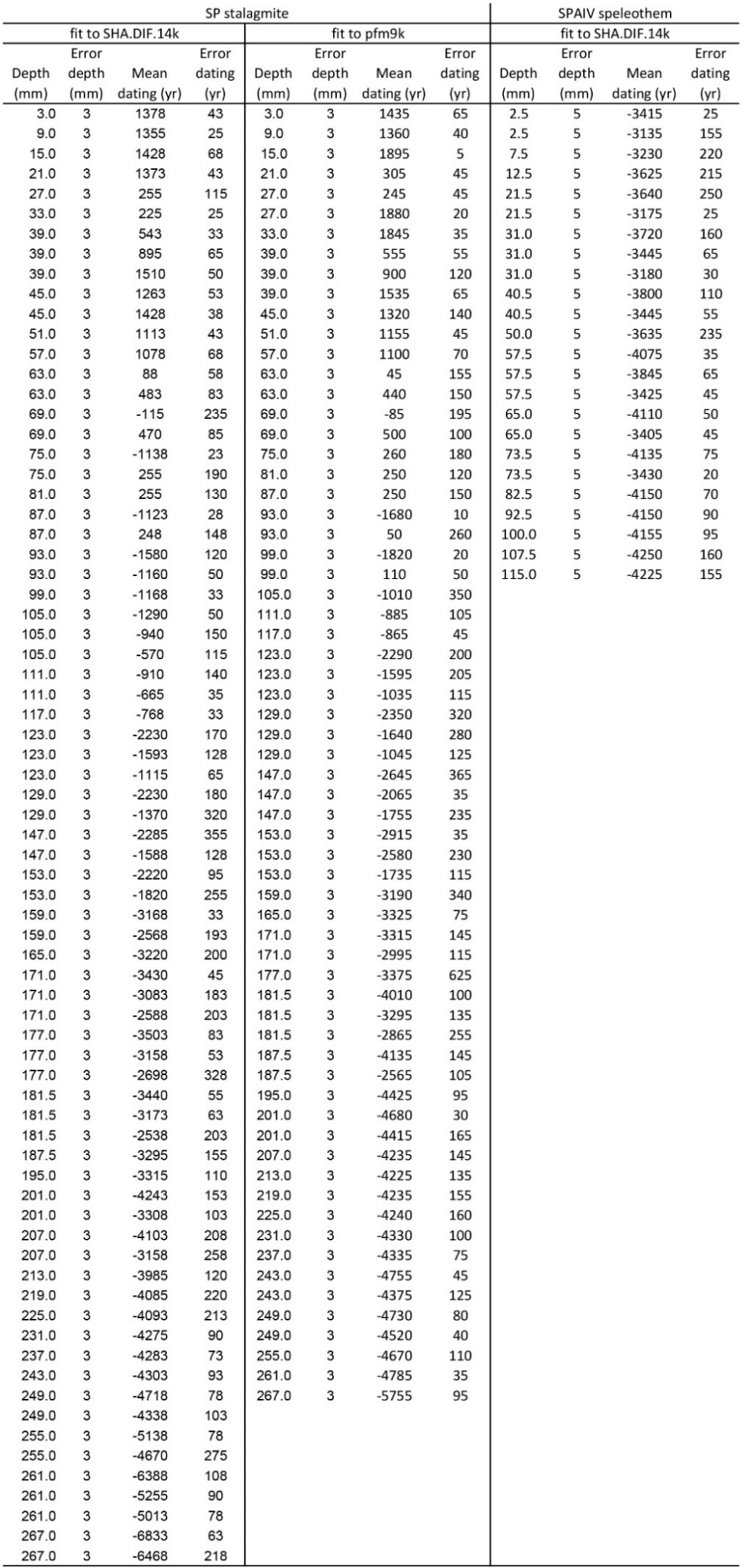
**

**Table S1.** Discrete dating intervals calculated by *archaeo_dating* software. Dating error is given at 95% of confidence.


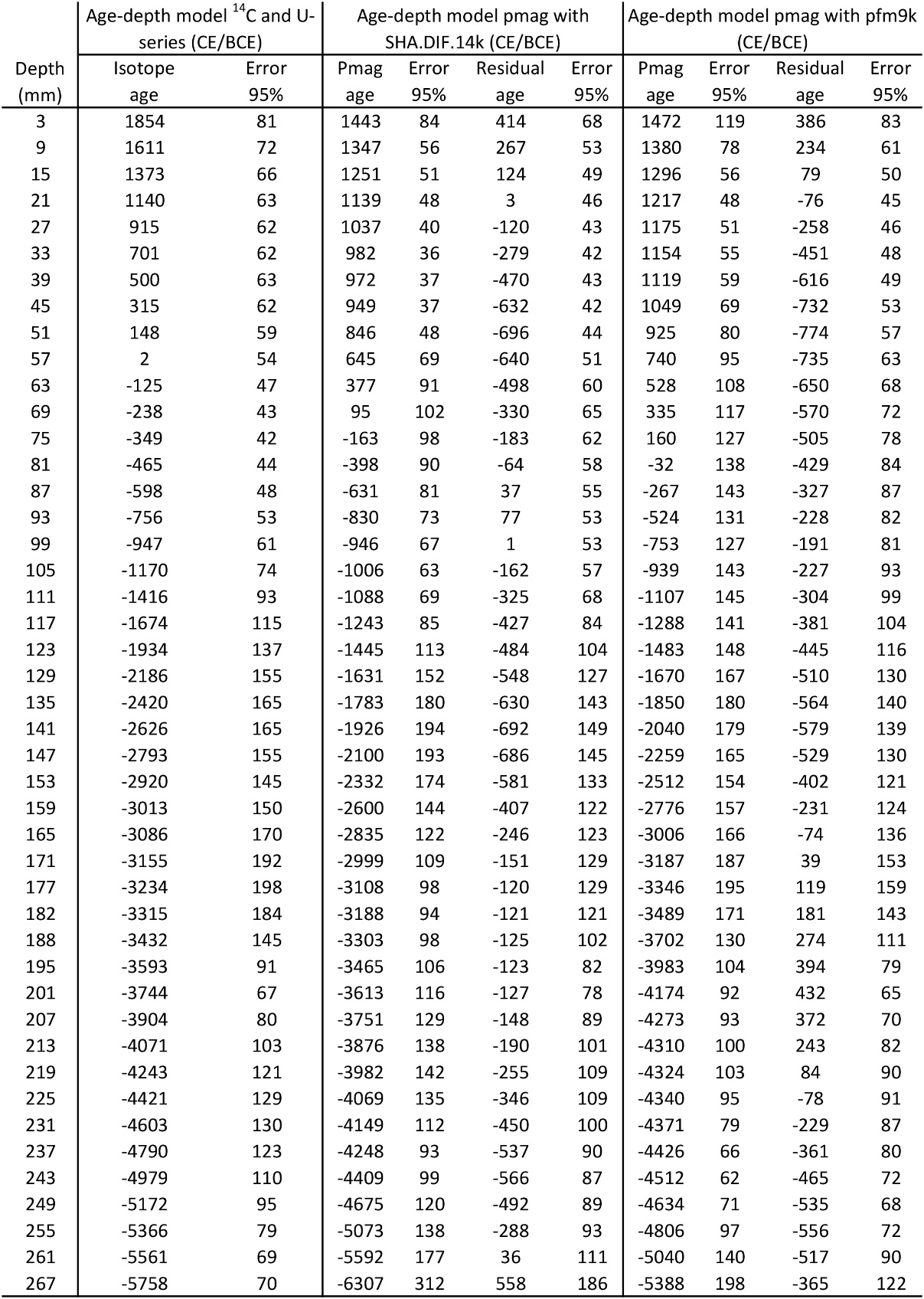


**Table S2.** Data corresponding to Figure 5A-B, consisting in the correlation between the age-depth model based on ^14^C and U-series dating and the models based on paleomagnetic data from SP and SPAIV stalagmites fitted to paleosecular variation (PSV) curves obtained from SHA.DIF.14k^4^ and pfm9k^5^ global reconstructions. CE: Common Era. BCE: Before Common Era. Depth: depth of the central point of each sample. Isotope age: Ages obtained by interpolation of ^14^C and U-series dating. Error 95%: 95% probability error. Pmag age: Ages obtained by statistical fitting of paleomagnetic data to PSV curves from the SHA.DIF.14k and pfm9k. Only the values corresponding to the depths where paleomagnetic directions were obtained are shown (in the complete analysis values were obtained every 1 mm).


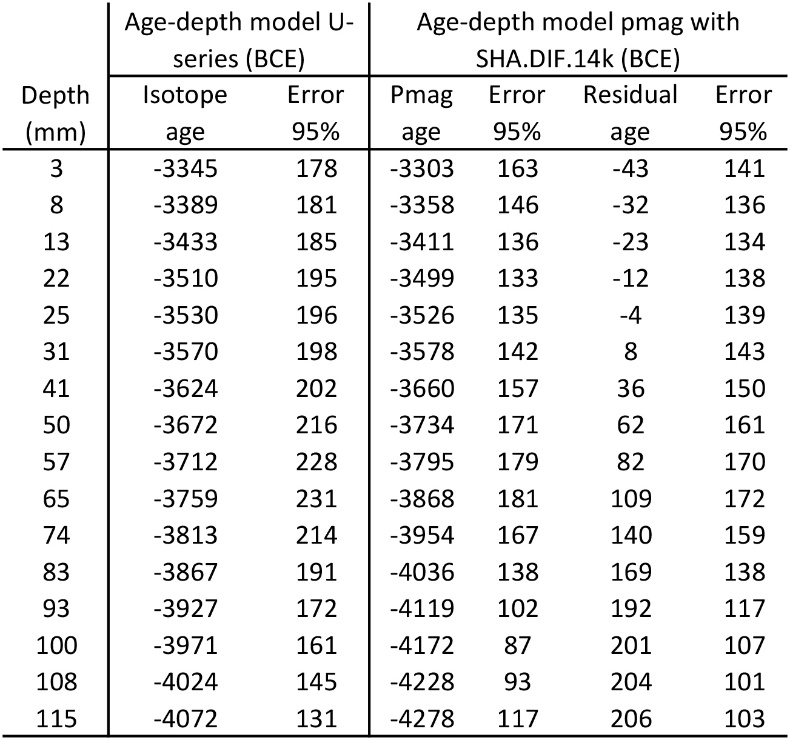


**Table S3.** Data corresponding to Fig. 5C, consisting in the correlation between the radiometric age-depth model and the paleosecular variation age model of the SPAIV speleothem fitted to paleosecular variation (PSV) curves obtained from SHA.DIF.14k global reconstruction^4^. CE: Common Era. Depth: depth of the central point of each sample. Isotope age: Age-depth model based on U-series is from Ponte et al. (2018). Error 95%: 95% probability error. Pmag age: Ages obtained by statistical fitting of paleomagnetic data to PSV curves from the SHA.DIF.14k and pfm9k^5^. Only the values corresponding to the depths where paleomagnetic directions were obtained are shown (in the complete analysis, values were obtained at 1 mm intervals).

**References**

1. Hua, G. *et al.* Trace and rare earth elemental geochemistry of carbonate succession in the Middle Gaoyuzhuang Formation, Pingquan Section: Implications for Early Mesoproterozoic ocean redox conditions. *J. Palaeogeogr.* **2**, 209–221 (2013).

2. Graven, H. *et al.* Compiled records of carbon isotopes in atmospheric CO2 for historical simulations in CMIP6. *Geosci. Model Dev.* **10**, 4405–4417 (2017).

3. Pavón-Carrasco, F. J., Rodríguez-González, J., Osete, M. L. & Torta, J. M. A Matlab tool for archaeomagnetic dating. *J. Archaeol. Sci.* **38**, 408–419 (2011).

4. Pavón-Carrasco, F. J., Osete, M. L., Torta, J. M. & De Santis, A. A geomagnetic field model for the Holocene based on archaeomagnetic and lava flow data. *Earth Planet. Sci. Lett.* **388**, 98–109 (2014).

5. Nilsson, A., Holme, R., Korte, M., Suttie, N. & Hill, M. Reconstructing holocene geomagnetic field variation: New methods, models and implications. *Geophys. J. Int.* **198**, 229–248 (2014).

6. Ponte, J. M., Font, E., Veiga-Pires, C. & Hillaire-Marcel, C. Speleothems as Magnetic Archives: Paleosecular Variation and a Relative Paleointensity Record From a Portuguese Speleothem. *Geochemistry, Geophys. Geosystems* **19**, 2962–2972 (2018).
